# Mobile Phone–Based Telemedicine Practice in Older Chinese Patients with Type 2 Diabetes Mellitus: Randomized Controlled Trial

**DOI:** 10.2196/10664

**Published:** 2019-01-04

**Authors:** Chenglin Sun, Lin Sun, Shugang Xi, Hong Zhang, Huan Wang, Yakun Feng, Yufeng Deng, Haimin Wang, Xianchao Xiao, Gang Wang, Yuan Gao, Guixia Wang

**Affiliations:** 1 Department of Endocrinology First Hospital of Jilin University Changchun China; 2 Health Management Center First Affiliated Hospital of Zhengzhou University Zhengzhou China

**Keywords:** telemedicine, type 2 diabetes, health management

## Abstract

**Background:**

Previous studies on telemedicine interventions have shown that older diabetic patients experience difficulty in using computers, which is a barrier to remote communication between medical teams and older diabetic patients. However, older people in China tend to find it easy to use mobile phones and personal messaging apps that have a user-friendly interface. Therefore, we designed a mobile health (mHealth) system for older people with diabetes that is based on mobile phones, has a streamlined operation interface, and incorporates maximum automation.

**Objective:**

The goal of the research was to investigate the use of mobile phone–based telemedicine apps for management of older Chinese patients with type 2 diabetes mellitus (T2DM). Variables of interest included efficacy and safety.

**Methods:**

A total of 91 older (aged over 65 years) patients with T2DM who presented to our department were randomly assigned to one of two groups. Patients in the intervention group (n=44) were provided glucometers capable of data transmission and received advice pertaining to medication, diet, and exercise via the mHealth telemedicine system. Patients assigned to the control group (n=47) received routine outpatient care with no additional intervention. Patients in both groups were followed up at regular 3-month intervals.

**Results:**

After 3 months, patients in the intervention group showed significant (*P*<.05) improvement in postprandial plasma glucose level. After 6 months, patients in the intervention group exhibited a decreasing trend in postprandial plasma glucose and glycated hemoglobin levels compared with the baseline and those in the control group (*P*<.05).

**Conclusions:**

Mobile phone–based telemedicine apps help improve glycemic control in older Chinese patients with T2DM.

**Trial Registration:**

China Clinical Trial Registration Center ChiCTR 1800015214; http://www.chictr.org.cn/showprojen.aspx?proj=25949 (Archived by WebCite at http://www.webcitation.org/73wKj1GMq).

## Introduction

Diabetes mellitus is one among the top three chronic, noninfectious diseases in the world [1]. Older people with diabetes constitute a high-risk group, and approximately one in five older patients with type 2 diabetes mellitus (T2DM) develops severe complications [2] such as diabetic neuropathy, nephropathy, retinopathy, or vasculopathy. Thus, these patients are susceptible to renal failure, loss of sight, loss of lower limbs [3], and the risk of severe hyperglycemia and hypoglycemia [4], which impairs their quality of life, imposes financial burden on the patients and community health care systems [2], and decreases life expectancy. Self-management of diabetes includes dietary monitoring, exercise, self-monitoring of blood glucose levels, and adjustments in mental status [5]. Telemedicine management systems allow for remote medical consultations and provision of personalized medical advice, including dietary and lifestyle-related advice from qualified care providers [6,7]. These systems offer an advantage for older patients as they help overcome the distance barrier and the loss of medical treatment opportunities [8,9]. Therefore, research on the applicability of telemedicine systems to older patients is of much clinical relevance.

A previously conducted computer-based telemedicine study [10] involving subjects who have had diabetes for more than 1 year showed that 6 months of telemedicine intervention obviously improved fasting and postprandial blood glucose (PBG) and triglyceride levels in the intervention group. However, as it was a computer-based study, we found in the course of the study that there were many older patients with diabetes who were unfamiliar with computer operations. In that study, a computer was designed to automatically transmit blood glucose meter readings. However, as the computer is not a portable device, transfer of information from the patient to the computer and then to the medical team was not found to be highly realistic [11]. The increasing popularity of mobile phones and user-friendly personal messaging apps has promoted their use in a subset of older patients [12]. We also found that older people in China are better at using mobile phones than computers. This type of special phenomenon is related to the economic development in China. The popularity of computers has occurred relatively late in China, and there are only a few older people who are familiar with computer operations. Nevertheless, with the rapid improvement in living standards in recent years, the popularity of mobile phones has maintained pace with that across the globe, and some older people have skipped the era of computer use. In fact, people are more open to learn to use a mobile phone than a computer, which requires a higher level of proficiency [13]. Considering the known obstacles in mobile phone use, we designed a mobile phone–based mobile health (mHealth) management platform to encourage mobile phone use by older patients; the user interface was designed to provide maximum possible simplicity and automation [14,15].

In this study, we conducted a mobile medical intervention experiment lasting for half a year to determine whether a diabetes mHealth management system based on mobile phones is suitable for older patients. We also evaluated the impact of using this system on glycemic control, treatment adherence, and the rate of occurrence of adverse events (for example, hypoglycemia), as well as overall satisfaction.

## Methods

### Ethical Considerations

The study protocol was designed in accordance with the Declaration of Helsinki and was approved by the ethics committee of the First Affiliated Hospital of Jilin University. This trial was registered at the China Clinical Trial Registration Center (ChiCTR 1800015214). Written informed consent was obtained from all patients prior to their enrollment.

### Participants and Recruitment

Patients who attended the outpatient endocrinology department of the First Affiliated Hospital of Jilin University between March and September 2016 were eligible for inclusion in this randomized controlled trial.

Inclusion criteria in this study were age older than 65 years, glycated hemoglobin (HbA_1c_) level 7.0% to 10.0%, and the ability to use a mobile phone. Exclusion criteria were illiteracy, abnormal liver and kidney function, severe diabetic complications, use of insulin pumps, and participation in other clinical trials.

A total of 91 patients were enrolled: 44 (19 males) in the intervention group and 47 (18 males) in the control group.

### Study Design and Randomization

Patients were randomly assigned to the intervention and control groups using the random number sequence generated by SPSS Statistics version 17.0 (IBM Corp) in batches of 6 patients at a time. Patients in the intervention group were provided training to independently use the mHealth management app and upload the glucometer data, which was then automatically transmitted to the medical server (glucometer was connected to the mobile phone via Bluetooth). The medical teams logged on to the system and sent medical advice and reminders to patients to monitor their glucose levels via the personal messaging app or telephonically every 2 weeks. Patients in the control group received a free glucometer and were followed up through conventional outpatient clinic appointments. For the control group patients, no limitations were imposed to the number of visits; however, they were instructed to monitor and record their blood glucose data regularly.

The study dietitian offered guidance for blood glucose monitoring and provided dietary advice based on the individual blood glucose levels. Patients in the intervention group used the app-based diet management software to input daily dietary intake. The dietitian received the daily dietary record of each patient via the mHealth app. On the basis of the analysis of this information, once-monthly dietary recommendations were sent from the dietitian to patients in the intervention group. The control group received dietary guidance from dietitians during face-to-face meetings at baseline and at the conclusion of all study-related procedures.

Information pertaining to physical activity (daily calorie expenditure) was obtained from patients in the intervention group via text message. The patients were instructed on how to text pedometer data to the study personnel. This information was analyzed, and each patient in the intervention group was provided with guidance related to aerobic and resistance-based exercises. In the control group, guidance related to exercise was provided during face-to-face dietary counseling session during clinic visits.

All patients were followed up in the outpatient clinic at 3-month intervals. Patients in both groups underwent physical examination, blood biochemical tests, follow-up clinic visits, and ambulatory therapy by the same medical team.

### Data Collection and Measurements

In order to assess the condition of patients, the following data were reviewed at baseline and every 3 months until the end of the experiment (a total of 3 times): medical history, treatment details, physical examination, and laboratory investigations. Patient compliance was assessed by the frequency of uploading blood glucose data in the intervention group. At the end of the experiment, all patients completed a satisfaction questionnaire, which contained 7 questions, with each question awarded a score of 1 and the highest possible total score being 7 points. Higher total score indicated better satisfaction.

### Statistical Analysis

Data were processed using SPSS Statistics version 17.0 (IBM Corp). Normally distributed variables are presented as mean and standard deviation; nonnormally distributed variables are presented as median and interquartile range. Between-group differences to normally distributed variables were assessed using an independent sample *t* test, whereas those to nonnormally distributed variables were assessed using a Mann-Whitney *U* test. For intragroup comparison, normally distributed variables were tested by paired *t* test and nonnormally distributed variables were tested by Wilcoxon rank-sum test. *P*<.05 was considered indicative of a statistically significant difference. Study figures were created using SigmaPlot (Systat Software Inc).

## Results

Baseline characteristics of the study population are summarized in Table 1. No significant between-group differences were observed with age, physical findings, or biochemical indices.

**Figure 1:**
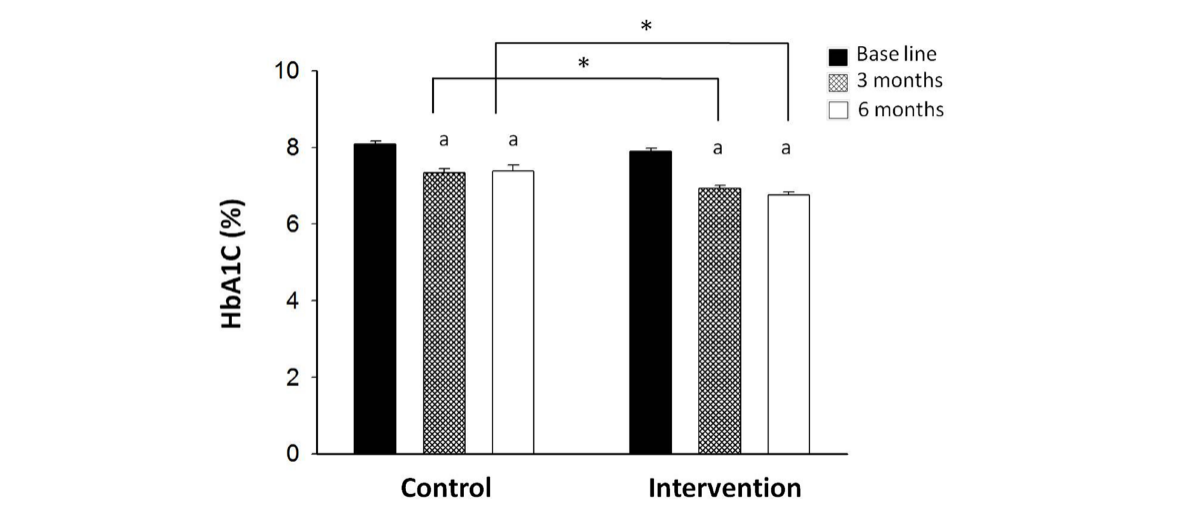
The changes in HbA_1c_ levels after follow-up in both groups. After 3 months, HbA_1c_ levels in both groups were significantly improved compared with baseline data (*P*<.01). Six months later, intervention group HbA_1c_ was lower than baseline (*P*<.01), as were the control group HbA_1c_ levels (*P*<.05). "a" indicates *P*<.05 versus baseline and asterisk indicates *P*<.05 versus control group.

**Figure 2:**
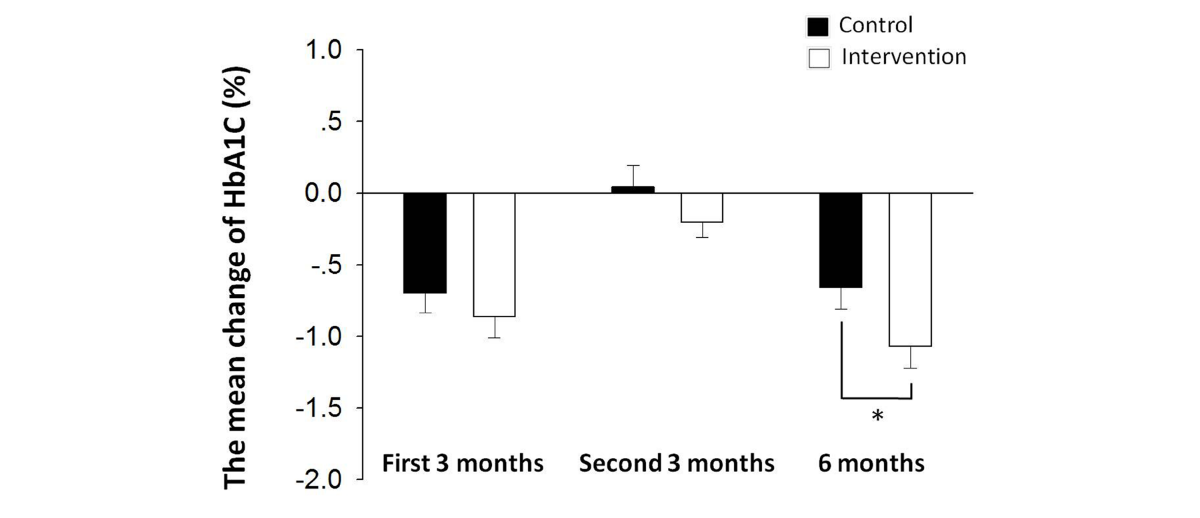
The comparison of the amplitude of change of HbA_1c_ levels in both groups. The mean change in HbA_1c_ levels from baseline to 6 months in the intervention group was significantly higher than that in the control group (*P*<.05). Asterisk indicates *P*<.05 versus control group.

**Figure 3:**
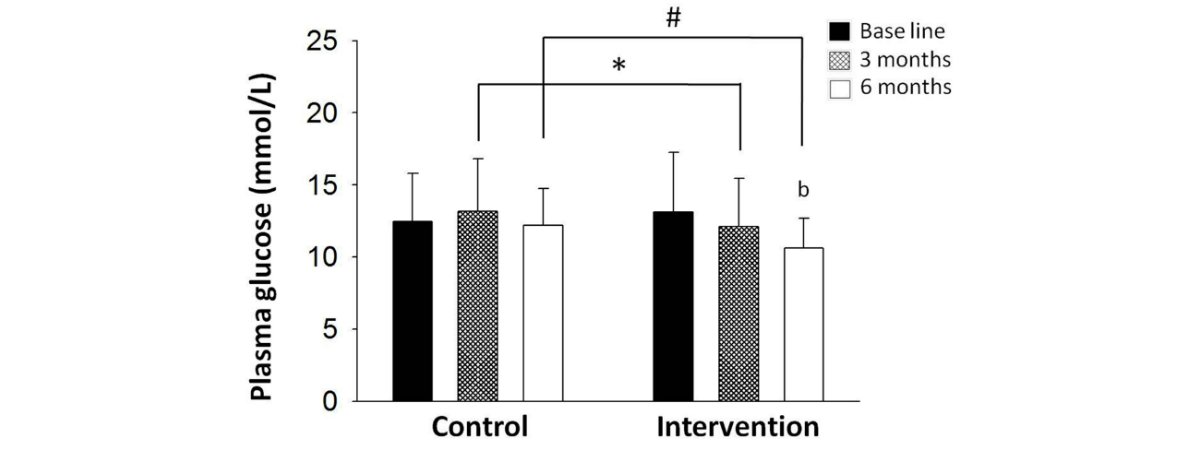
The changes in postprandial blood glucose levels after follow-up in both groups. At the end of 3 and 6 months, the intervention group postprandial blood glucose was significantly lower than the control group postprandial blood glucose (*P*<.05 and *P*<.01). "b" indicates *P*<.01 versus baseline; asterisk indicates *P*<.05; and # indicates *P*<.01 versus control group.

HbA_1c_ level reflects the level of glycemic control over the past 3 months. After the first 3 months, we noted a significant improvement in HbA_1c_ levels over the baseline level in both the control group (7.18% [SD 0.85%] vs 7.88% [SD 0.64%], *P*<.001; Table 2 and Figure 1) and the intervention group (6.97% [SD 0.65%] vs 7.84% [SD 0.73%], *P*<.001; Table 2 and Figure 1). Since patients in both groups were given medication, diet, and exercise guidance at the beginning of the trial, a reduction in HbA_1c_ level was observed in both groups at the completion of 3 months, and there was no significant difference between the two groups (*P*=.25; Table 2 and Figure 2). Patients in the intervention group exhibited a decrease in PBG levels relative to baseline; a significant between-group difference was observed in this respect (*P*=.04; Table 2 and Figure 3).

At 6 months, the HbA_1c_ level in the intervention group was significantly lower than that at baseline (6.84% [SD 0.765%] vs 7.84% [SD 0.73%], *P*<.001; Figure 1) and that in the control group at 6 months (6.84% [SD 0.765%] vs 7.22% [SD 0.87%], *P*=.02; Table 2 and Figure 1). The extent of decrease in HbA_1c_ level from baseline level in the intervention group was more than that in the control group (1.07% [SD 0.89%] vs 0.62% [SD 1.00%], *P*=.045; Figure 2). After the 6 months, PBG levels in the intervention group demonstrated continuous improvement as compared with baseline level (10.62 [SD 2.07] mmol/L vs 13.10 [SD 4.13] mmol/L, *P*=.002; Figure 3) and that at 3 months (10.62 [SD 2.07] mmol/L vs 12.09 [SD 3.35] mmol/L, *P*=.03; Table 2 and Figure 3) and were also significantly lower than that in the control group at 6 months (10.62 [SD2.07] mmol/L vs 12.19 [SD 2.54] mmol/L, *P*=.004; Table 2 and Figure 3).

After 6 months, we obtained satisfactory results from the survey of patients in the intervention group. Higher total scores indicated better satisfaction. The average satisfaction score was 6.3 (SD 0.78). Individual questions measured details regarding whether the intervention improved the self-monitoring of patients’ blood glucose levels (0.93 [SD 0.14]), diet, exercise and other self-management skills (0.85 [SD 0.20]), and knowledge of diabetes (0.98 [SD 0.08]), as well as the effect on their psychological status (0.96 [SD 0.12]; Multimedia Appendix 1).

**Table 1:** Baseline characteristics of the two groups.

Characteristic	Control (n=47)	Intervention (n=44)	*P* value
Age in years, median (IQR)^a^	68.04 (66-72)	67.9 (66-71)	.85
Gender, male, n (%)	18 (38)	19 (43)	—^b^
Diabetes mellitus, duration in years, mean (SD)	11.52 (7.73)	11.19 (6.39)	.80
FBG^c^ (mmol/L), mean (SD)	7.78 (1.85)	8.0 (2.54)	.41
PBG^d^ (mmol/L), mean (SD)	12.44 (3.37)	13.10 (4.13)	.46
HbA_1c_^e^ (%), mean (SD)	7.88 (0.64)	7.84 (0.73)	.53
TC^f^ (mmol/L), mean (SD)	4.92 (1.24)	5.00 (0.97)	.76
TG^g^ (mmol/L), mean (SD)	2.31 (1.85)	2.41 (1.82)	.80
HDL-C^h^ (mmol/L), median (IQR)	1.21 (1.05-1.40)	1.09 (0.85-1.25)	.28
LDL-C^i^ (mmol/L), median (IQR)	2.86 (2.28-3.67)	2.92 (2.37-3.29)	.84
BUN^j^ (mmol/L), median (IQR)	5.79 (4.76-6.69)	5.62 (5.13-7.05)	.39
Cr^k^ (mmol/L), median (IQR)	59.1 (52.58-69.98)	65.05 (54.28-76.58)	.26
AST^l^ (U/L), median (IQR)	21.00 (17.50-24.00)	21.30 (17.75-24.25)	.53
ALT^m^ (U/L), median (IQR)	20.00 (13.00-32.25)	20.50 (14.70-30.00)	.83
r-GT^n^ (U/L), median (IQR)	20.00 (16.00-26.75)	24.5 (19.00-36.00)	.80
Body mass index, median (IQR)	23.30 (21.93-25.88)	23.60 (22.48-26.38)	.63
Blood pressure (mm Hg), systolic, mean (SD)	136.04 (19.37)	132.55 (11.82)	.55
Blood pressure (mm Hg), diastolic, median (IQR)	80.00 (73.50-90.00)	83.00 (74.00-87.75)	.99

^a^IQR: interquartile range.

^b^Indicates a range of values.

^c^FBG: fasting blood glucose.

^d^PBG: postprandial blood glucose.

^e^HbA_1c_: glycated hemoglobin.

^f^TC: total cholesterol.

^g^TG: triglyceride.

^h^HDL-C: high-density lipoprotein–cholesterol.

^i^LDL-C: low-density lipoprotein–cholesterol.

^j^BUN: blood urea nitrogen.

^k^Cr: creatinine.

^l^AST: aspertate aminotransferase.

^m^ALT: alanine aminotransferase.

^n^r-GT: r-glutamyltransferase.

**Table 2:** The follow-up data of the two groups.

Characteristics	3 months			6 months		
	Control	Intervention	*P* value	Control	Intervention	*P* value
FBG^a^ (mmol/L), mean (SD)	7.57 (2.15)	7.20 (1.70)	.41	7.24 (2.49)	7.26 (2.17)	.96
PBG^b^ (mmol/L), mean (SD)	13.15 (3.64)	12.09 (3.35)	.04	12.19 (2.54)	10.62 (2.07)^c^	.004
HbA_1c_^d^ (%), mean (SD)	7.18 (0.85)^e^	6.97 (0.65)^e^	.25	7.22 (0.87)	6.84 (0.76)^e^	.02
TC^f^ (mmol/L), mean (SD)	4.84 (1.08)	4.94 (0.80)	.57	4.66 (1.19)	4.63 (0.70)	.88
TG^g^ (mmol/L), mean (SD)	1.69 (0.97)	1.66 (0.84)^e^	.86	1.75 (0.86)	1.79 (0.87)	.80
HDL-C^h^ (mmol/L), median (IQR^i^)	1.34 (1.12-1.51)	1.30 (1.07-1.45)	.39	1.30 (1.15-1.49)	1.2 (1.02-1.35)	.46
LDL-C^j^ (mmol/L), median (IQR)	2.99 (2.08-3.52)	2.87 (2.64-3.27)	.56	2.85 (2.03-3.61)	2.88 (2.43-3.14)	.68
BMI^k^, median (IQR)	23.25 (22.13-26.23)	23 (22.68-27.43)	.07	22.62 (21.55-24.45)	23.8 (22.5-27.3)	.30
Blood pressure (mm Hg), systolic, mean (SD)	140.61 (14.433)	137.05 (15.07)	.40	130.69 (11.22)	134.48 (9.08)	.22
Blood pressure (mm Hg), diastolic, median (IQR)	80 (69-86.75)	79 (73.75-84.25)	.86	79 (75-84)	80 (78-84)	.78

^a^FBG: fasting blood glucose.

^b^PBG: postprandial blood glucose.

^c^*P*<.01 versus baseline.

^d^HbA_1c_: glycated hemoglobin.

^e^*P*<.05.

^f^TC: total cholesterol.

^g^TG: triglyceride.

^h^HDL-C: high-density lipoprotein–cholesterol.

^i^IQR: interquartile range.

^j^LDL-C: low-density-lipoprotein–cholesterol.

^k^BMI: body mass index.

## Discussion

### Principal Findings

We completed a 6-month, prospective, randomized controlled trial of the mHealth telemedicine system in patients with T2DM aged over 65 years. The results showed that the PBG and HbA_1c_ levels in the intervention group were significantly lower than those in the control group; our results are very similar to those reported by Lim et al [16] and Egede et al [17]. At the completion of 3 months, the PBG level in the intervention group was 1.02 mmol/L lower than that at baseline; by the completion of 6 months, the PBG levels showed a progressive decrease of approximately 1.21 mmol/L relative to that at the completion of 3 months. After 6 months, we also observed a significant difference between the intervention and control groups in this respect. At the completion of 3 months, the HbA_1c_ level in the intervention group had decreased by 1% as against 0.66% in the control group. Although the between-group difference was not statistically significant, the HbA_1c_ levels in the intervention group at 6 months showed a further decline of 0.13% as against an increase of 0.04% in the control group. After the sixth month, the intervention group showed continuous improvement in HbA_1c_ levels and the between-group difference was statistically significant; this finding is consistent with the results of Cho et al [18], who also demonstrated the efficacy of telemedicine interventions after a certain period of time. These findings suggest that older patients require time to familiarize themselves with the mHealth system. However, after self-training and remote support from the medical team, the patients started independent use of the portable smart device, which reflected in the positive effects [19]. In this research, both groups showed improved blood glucose and HbA_1c_ levels. This may be attributable to personalized medicine and dietary and exercise plans provided to all subjects [20]. However, without remote supervision and ongoing support, it is difficult to achieve sustained efficacy in the long term; thus, long-term follow-up is essential for older patients with diabetes [21]. During the study, 13 subjects in the control group (7 with hyperglycemia and 6 with hypoglycemia) and 5 subjects in the intervention group (4 with hyperglycemia and 1 with hypoglycemia) required adjustment of drug dosage for titration of glycemic control; however, none of the patients in either group experienced any serious adverse events or aggravation of complications. The intervention group had significantly fewer hypoglycemic events compared with the control group. This was likely attributable to prompt identification of the risk of hypoglycemia in the intervention group by the medical team via the mHealth platform and the consequent implementation of timely corrective actions [10,22,23]. After the trial, over 89% of patients in the intervention group continued to measure their blood glucose level 2 to 3 days each week. Intervention group satisfaction survey responses indicated that frequent communication with the medical team via the mHealth platform enhanced patient understanding of diabetes, increased their awareness, and helped alleviate depressive symptoms [8,24].

### Comparison With Prior Work

Previous studies have shown that telemedicine interventions can improve blood glucose control in patients with diabetes [25,26]; however, the applicability of telemedicine for older patients has rarely been discussed.

The study by Quinn et al [24] and Kim et al [27] showed that middle-aged and older patients with diabetes have good interaction in a mobile phone–based diabetes education environment and that it significantly improves the self-management of blood glucose levels. However, the study was conducted over a period of 1 month, which is too short to determine the compliance of older patients over the long term with remote intervention. However, Egede et al [17] conducted a 12-month-long study involving remote psychotherapy intervention for older diabetic patients. Older diabetic patients not only maintained good compliance but also achieved long-term glycemic control. However, the intervention involved only psychotherapy, and there was no routine medication-, diet- and exercise-related intervention. Therefore, the study was not designed to determine the advantages of remote intervention with regular outpatient treatment. Cho et al [18] performed a 6-month comparative study of telemedicine and traditional outpatient treatment; although the benefits of telemedicine were not found at 3 months, HbA_1c_ levels were significantly improved at 6 months and the benefit was mainly found among women aged over 40 years. Williams et al [28] conducted a 12-month long-distance interventional clinical trial among African Americans aged over 21 years; the results suggested that long-term remote interventions can improve long-term glycemic control. However, all the above studies involved remote interventions in a wide range of age groups. Since the cognitive ability of older patients is relatively low, the operation interface used by middle-aged patients cannot be expected to be equally effective in older patients. Therefore, we greatly simplified the user interface of our telemedicine system to make it suitable for use by older patients. This enhanced the confidence of patients and their ability to follow the advice and provided us with valuable data that can be analyzed.

### Limitations

In general, telemedicine facilitates good glycemic control in older diabetic patients. In this study, the personal and family medical history, smoking history, history of alcohol intake, birth history, history of drug allergy, and personal living environment were not included in the analysis [6]. However, these factors can potentially affect the nutritional status and function of major organs; in addition, this information is important for the assessment of the quality of life of patients [29,30]. Moreover, data collected from dietary caloric intake and expenditure are not as accurate as blood glucose data; therefore, the effect of dietary and exercise-related guidance on glycemic control was not reliably measured; it is necessary to develop an accurate data collection method for calorie intake and consumption [31]. When this was achieved, telemedicine assisted the medical team and allowed the team to provide timely warnings of the risk of hypoglycemia or hyperglycemia as well as encouraged patients to continue their diet and exercise plan.

### Conclusions

In this study, PBG level in the intervention group was significantly lower than that in the control group after the first 3 months. The improvement in glycemic control was sustained after 6 months and showed a significant difference from that in the control group. Our results suggest that the improved glycemic control in the intervention group was attributable to improved communication between doctors and patients with real-time tracking of older diabetic patients by the mHealth system and improved patient compliance after implementation of mHealth monitoring. On the basis of our findings, we can conclude that telemedicine is effective and safe for older diabetic patients.

## Appendix

Multimedia Appendix 1 Satisfaction survey.

Multimedia Appendix 2 CONSORT‐EHEALTH checklist (V 1.6.1).

## Abbreviations

HbA_1c_: glycated hemoglobin
PBG: postprandial blood glucose
T2DM: type 2 diabetes mellitus
