# Supplementary material for: Mobile Phone–Based Telemedicine Practice in Older Chinese Patients with Type 2 Diabetes Mellitus: Randomized Controlled Trial
Source: JMIR Mhealth Uhealth. 2019 Jan 4;7(1):e10664. doi: 10.2196/10664 (PMC6682265; doi:10.2196/10664)
Supplement: Multimedia Appendix 1 [file mhealth_v7i1e10664_app1.pdf]

Multimedia Appendix 1. Satisfaction survey.

Question		Score					mean±SD
1. Are you satisfied with current treatment?	Dissatisfied	0.	0.3	0.6	1.0	Very satisfied	0.89±0.19
2. Is it convenient for you receive telemedical management?	Inconvenient	0.	0.3	0.6	1.0	Very convenient	0.81±0.20
3. Is it helpful to self-monitoring of blood glucose?	Not helpful.	0.	0.3	0.6	1.0	Very helpful	0.93±0.14
4. Is it helpful adhere to diet and exercise management?	Not helpful.	0.	0.3	0.6	1.0	Very helpful	0.85±0.20
5. Is telemedical management helpful for the knowledge of diabetes?	Not helpful.	0.	0.3	0.6	1.0	Very helpful	0.98±0.08
6. Is telemedical management helpful to relieve Mental distress?	Not relieved.	0.	0.3	0.6	1.0	Very relieved	0.96±0.12
7. Will you recommend telemedical management to others?	No.	0.	0.3	0.6	1.0	Yes. Will recommend actively	0.91±0.02
